# Femoroazetabuläres Impingement beim Jugendlichen und Adoleszenten

**DOI:** 10.1007/s00117-023-01197-6

**Published:** 2023-09-12

**Authors:** Iris-M. Noebauer-Huhmann, Felix R. M. Koenig, Catharina Chiari, Florian Schmaranzer

**Affiliations:** 1 Universitätsklinik für Radiologie und Nuklearmedizin, Abteilung für Neuroradiologie und Muskuloskelettale Radiologie, Medizinische Universität Wien, Wien, Österreich; 2 Universitätsklinik für Orthopädie und Unfallchirurgie, Klinische Abteilung für Orthopädie, Medizinische Universität Wien, Wien, Österreich; 3 Abteilung für Kinderorthopädie und Fußchirurgie, Orthopädisches Spital Speising, Wien, Österreich; 4 Universitätsklinik für Diagnostische‑, Interventionelle- und Pädiatrische Radiologie, Inselspital Bern, Universität Bern, Bern, Schweiz

**Keywords:** Hüftgelenk, Schmerzsyndrom, Projektionsröntgenaufnahme, Magnetresonanztomographie, Femurtorsion, Hip, Pain, Diagnostic X‑ray radiology, Magnetic resonance imaging, Femoral torsion

## Abstract

Das femoroazetabuläre Impingement-Syndrom (FAIS) wird durch einen repetitiven mechanischen Konflikt zwischen Azetabulum und proximalem Femur insbesondere bei Flexion und Innenrotation hervorgerufen. Beim femoroazetabulären Impingement (FAI) vom Cam-Typ bewirkt eine Asphärizität am femoralen Kopf-Hals-Übergang die Induktion von Scherkräften am Azetabulum. Beim Pincer-Typ kann eine Retroversion der Pfanne und/oder eine vermehrte Überdachung vorliegen. Ein wichtiger mechanischer Einflussfaktor, welcher ein Impingement oder auch Hüftinstabilität verstärken oder kompensieren kann, ist die Femurtorsion. Meistens treten Torsionsstörungen kombiniert mit anderen ossären Deformitäten auf. Zu beachten ist, dass ein hoher Prozentsatz der Adoleszenten mit knöchernen FAI-Morphologien asymptomatisch bleibt. Die Diagnose des FAIS wird daher klinisch gestellt, die Bildgebung zeigt die zugrundeliegende Morphologie. Primäre Bildgebung ist das Röntgenbild in 2 Ebenen zur Beurteilung der Hüftgelenküberdachung und der azetabulären Version. Die vollständige Zirkumferenz des Femurs ist jedoch nur in der Magnetresonanztomographie (MRT) beurteilbar, ebenso Läsionen des Labrums und Knorpels sowie des Knochenmarks und der umgebenden Weichteile. Das MRT-Protokoll sollte routinemäßig eine Bestimmung der Rotation des Femurs beinhalten. Zudem sollten flüssigkeitssensitive Sequenzen des Beckens zum groben Ausschluss degenerativer oder entzündlicher extraartikulärer Veränderungen akquiriert werden.

## Abstract (en)

Femoroacetabular impingement syndrome (FAIS) is caused by a repetitive mechanical conflict between the acetabulum and the proximal femur, occurring in flexion and internal rotation. In cam impingement, bony prominences of the femoral head–neck junction induce chondrolabral damage. The acetabular type of FAIS, termed pincer FAIS, may be either due to focal or global retroversion and/or acetabular overcoverage. Combinations of cam and pincer morphology are common. Pathological femoral torsion may aggravate or decrease the mechanical conflict in FAI but can also occur in isolation. Of note, a high percentage of adolescents with FAI-like shape changes remain asymptomatic. The diagnosis of FAIS is therefore made clinically, whereas imaging reveals the underlying morphology. X‑rays in two planes remain the primary imaging modality, the exact evaluation of the osseous deformities of the femur and chondrolabral damage is assessed by magnetic resonance imaging (MRI). Acetabular coverage and version are primarily assessed on radiographs. Evaluation of the entire circumference of the proximal femur warrants MRI which is further used in the assessment of chondrolabral lesions, and also bone marrow and adjacent soft tissue abnormalities. The MRI protocol should routinely include measurements of femoral torsion. Fluid-sensitive sequences should be acquired to rule out degenerative or inflammatory extra-articular changes.

Das femoroazetabuläre Impingement (FAI) stellt klinisch ein schmerzhaftes Syndrom des Hüftgelenks dar (daher auch femoroazetabuläres Impingement-Syndrom, FAIS). Es tritt durch einen repetitiven mechanischen Konflikt zwischen Azetabulum und proximalem Femur auf, typischerweise in kombinierter Hüftflexion und Innenrotation (vorderer Impingement-Test). Morphologisch ursächlich für einen pathologischen knöchernen Kontakt kann entweder eine Taillierungsstörung des Femurs (Cam) oder eine Mehrüberdachung und/oder Retroversion des Azetabulums (Pincer) sein; in vielen Fällen liegen auch Mischformen vor. Einen wichtigen Einflussfaktor stellt die Femurtorsion dar, die ursächlich, verstärkend oder kompensierend wirken kann.

Je nach körperlicher Aktivität können diese Impingement-Morphologien bereits in der Adoleszenz zu Hüftschmerzen führen [1]. Allerdings weisen auch viele asymptomatische Jugendliche und junge Erwachsene eine FAI-Morphologie auf: Ein Cam-Impingement wurde bei 16,8–26,5 %, ein Pincer-Impingement bei 17,6–32,4 % und eine Mischform bei 4,9– 6,1 % der Jugendlichen beschrieben [2, 3]. Die Diagnose des Impingement-Syndroms wird daher klinisch gestellt [1]. Ziel der gelenkerhaltenden Chirurgie ist die Wiederherstellung der Hüftgelenkfunktion, Schmerzreduktion und Verzögerung der Arthroseprogression mittels Korrektur der zugrundeliegenden ossären Deformitäten [4, 5].

## Cam-Impingement

Das Cam-Impingement ist durch eine verminderte Taillierung des Übergangs des Hüftkopfes zum Schenkelhals gekennzeichnet, typischerweise anterosuperior (mit punctum maximum bei 1 Uhr). Die Aspherizität führt dazu, dass der Femur in das Azetabulum gleitet und Scherkräfte sowie Schäden an der chondrolabralen Übergangszone induziert (*Inklusions-Impingement*). Die Therapie der Wahl ist abhängig von der Ausdehnung der Cam-Deformität eine arthroskopische oder offene femorale Osteochondroplastie mit dem Ziel der Wiederherstellung der physiologischerweise konkaven Schenkelhalskontur [1].

**Figure 1:**
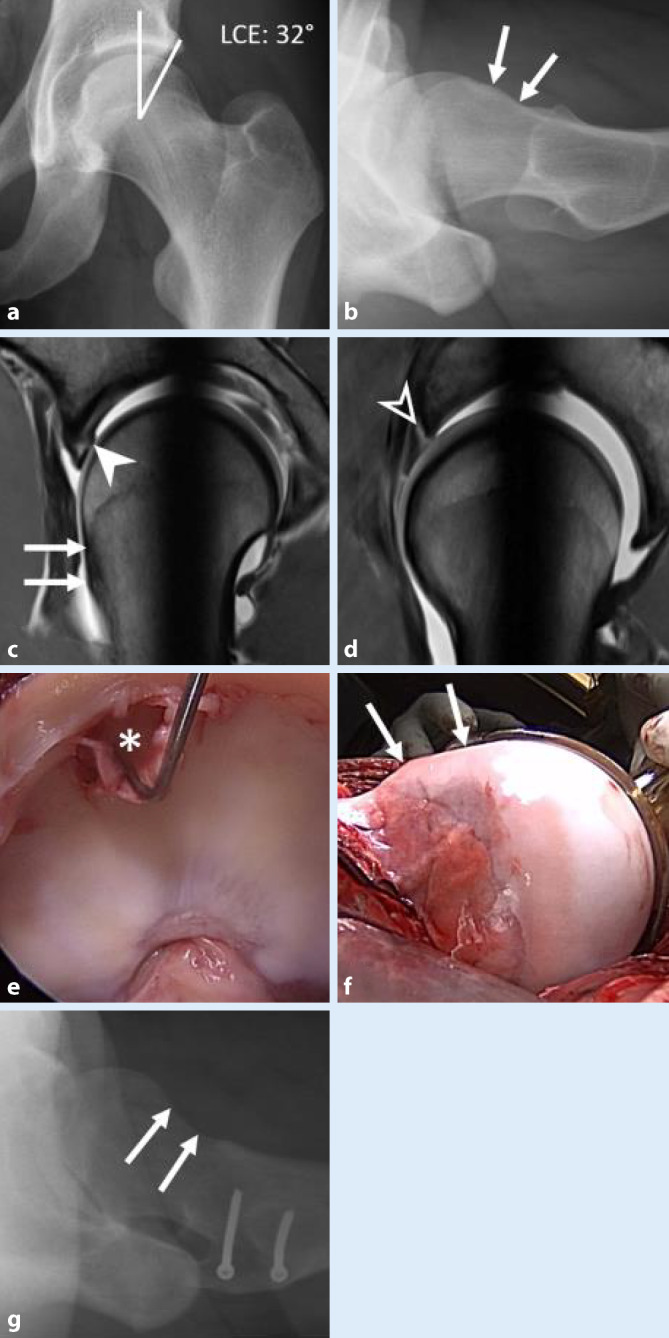

**Figure 2:**
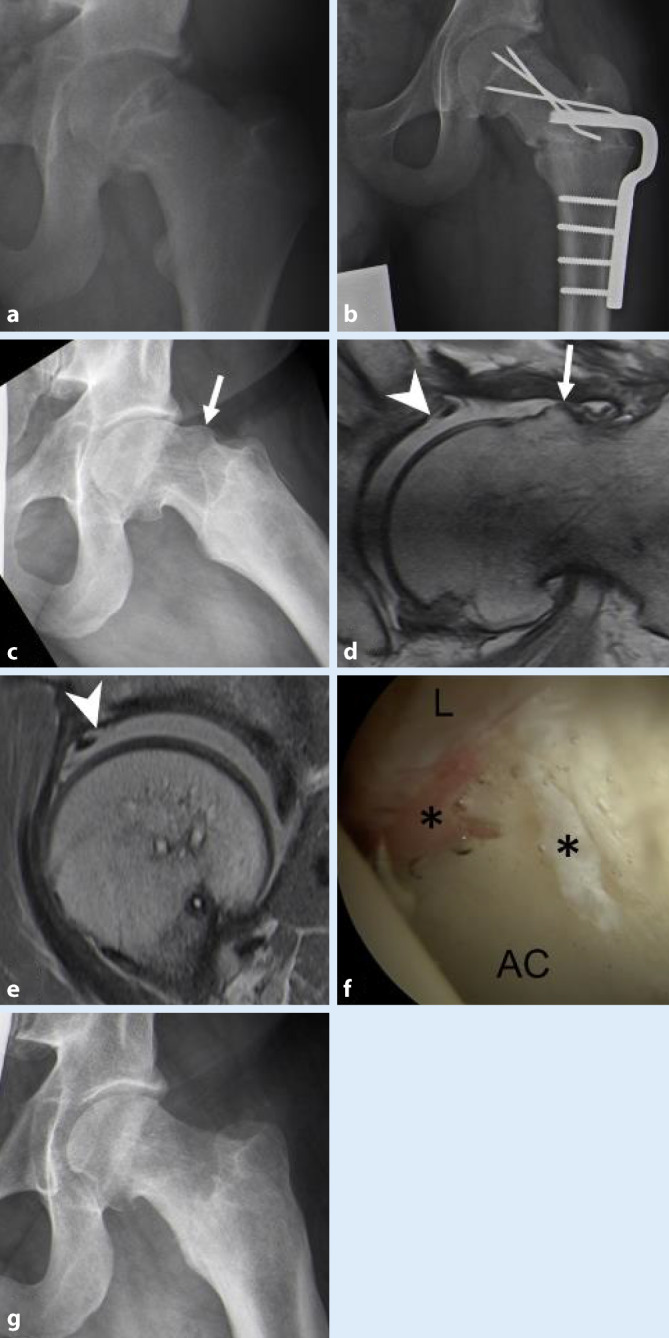

In der Genese der sog. *primären (idiopathischen) Cam-Morphologie* (Abb. 1) gibt es zunehmende Evidenz, dass Stop-and-Go-Sportarten (Fußball, Hockey, Basketball) während des Wachstumsschubs zu einer pathologischen Belastung der Epiphysenfuge führen [6]. Die pathologische Belastung der Wachstumsfuge führt zu einer Hypertrophie oder Extension der Epiphyse [7], möglicherweise als Reaktion im Sinne eines Stabilisierungsversuchs gegenüber juxtaphysären Mikrotraumata. Die Deformierung findet bei Überbelastung meist bis zum Physenschluss statt [8]. Zudem scheinen in der Genese der primären Cam-Morphologie genetische Faktoren eine Rolle zu spielen, da Geschwister ein 2,8fach erhöhtes Risiko für die Entwicklung eines Cam-Impingements und ein 2faches Risiko für ein Pincer-Impingement haben [9]. Davon zu unterscheiden sind die sog. *sekundären Cam-Deformitäten, *die nach manifester oder subklinischer Epiphyseolysis capitis femoris (ECF; *engl.:* „slipped capital femoral epiphysis“, SCFE; Abb. 2), Morbus Perthes, Perthes-like-Deformitäten nach kongenitaler Hüftdysplasie/septischer Arthritis oder proximalen Femurfrakturen entstehen.

Die *Post-slip-Cam-Deformität* stellt eine besonders schwere Form dar, da durch die dorsale Abkippung der Epiphyse, die weiterhin mit dem Azetabulum artikuliert, in vielen Fällen eine Retrotorsion des Femurs sowie eine Varusstellung (CCD-Winkel < 125°) des Schenkelhalses resultiert und das vordere Impingement zusätzlich verstärkt [10].

## Pincer-Impingement

Beim Pincer-Impingement (*Beißzangen-Impingement*) besteht eine zu starke Ausbildung des azetabulären Erkers mit übermäßiger Überdachung des Hüftkopfes und/oder eine reduzierte Anteversion der Hüftpfanne. Im Gegensatz zum Cam-Impingement kann der Femurkopf nicht ins Azetabulum gleiten, und es kommt stattdessen zu einer direkten Krafteinwirkung (*Impaktions-Impingement*) am Azetabulum mit einer zirkumferenziellen Degeneration und Ossifikation des Labrums und einer linearen Knorpelschädigung. Je nach Ausmaß der Deformität reicht die chirurgische Therapie vom Zurückfräsen des Azetabulums mit konsekutiver Labrumrefixation bis hin zur einer antevertierenden periazetabulären Osteotomie zur Therapie der schweren azetabulären Retroversion bei jungem Patienten mit gutem Knorpelstatus [11].

## Bedeutung der femoralen Torsion

**Figure 3:**
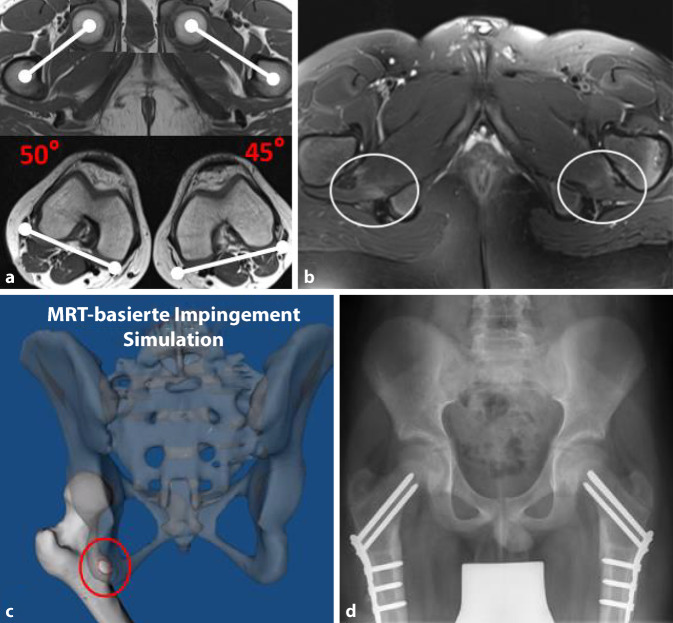

In den letzten Jahren hat die Integration der Femurtorsion in das pathomechanische Konzept des FAIS und der Hüftgelenkinstabilität zu neuen Erkenntnissen in der Diagnostik und Behandlung von Torsionspathologien geführt. Die Femurtorsion beschreibt die Stellung des proximalen Femurs relativ zur femoralen Kondylenachse in der sagittalen Ebene und beeinflusst so den Bewegungsspielraum im Hüftgelenk. Eine vermehrte Femurtorsion erhöht die Innenrotation/schränkt die Außenrotation der Hüfte ein und vice versa für die reduzierte Femurtorsion (Retrotorsion; [12]). Somit prädisponiert die Retrotorsion zu einem vorderen intraartikulären Impingement – unter Umständen auch ohne Vorliegen einer Cam-Deformität – sowie zu einem extraartikulärem Kontakt zwischen distalem Schenkelhals und Spina iliaca anterior inferior [13]. Eine exzessiv vermehrte femorale Antetorsion wiederum kann zu einem Einwärtsgang und einem posterioren extraartikulären Impingement zwischen intertrochantärem Massiv/Trochanter minor und Tuber ischiadicum führen. Dies führt typischerweise zur Einengung des Ischiofemoralraums mit konsekutivem Ödem im M. quadratus femoris [14]. Es konnte gezeigt werden, dass Torsionsstörungen je nach Patientenkollektiv bei bis zu 1 von 6 Patienten, die für einen gelenkerhaltenden hüftchirurgischen Eingriff abgeklärt werden, vorliegen [15]. Je nach Schweregrad der Torsionsstörung bzw. dem Vorliegen assoziierter Deformitäten bedarf es in ausgewählten Fällen einer derotierenden oder rotierenden Femurosteotomie ([16, 17]; Abb. 3). Wichtig zu beachten ist auch, dass die Femurtorsion während der Skelettreifung physiologischerweise abnimmt und sich eine pathologische Femurtorsion in den ersten beiden Lebensdekaden bis zum Physenschluss sogar normalisieren kann [18].

## Bildgebende diagnostische Modalitäten in der FAI-Abklärung

### Projektionsröntgenaufnahme

Standard der initialen Bildgebung ist nach wie vor eine Röntgenaufnahme in zumindest 2 Ebenen; neben der Darstellung einer potenziellen FAI-Morphologie dient sie dem Ausschluss anderer Pathologien, wie z. B. Entzündungen, Hüftkopfnekrosen oder Frakturen [19]. Die Röntgenuntersuchung umfasst initial eine Beckenübersicht a.-p. und eine axiale Aufnahme.

Die Beckenübersichtsaufnahme erfolgt üblicherweise zunächst im Liegen. Hier liegen die verlässlichsten Referenzwerte zur Prognose vor, gleichzeitig ist diese Position mit späteren intraoperativen Aufnahmen vergleichbar [20]. Durchführung: Rückenlage, 15° innenrotierte Beine, Film-Fokus-Abstand 120 cm.

Die Wahl der axialen Aufnahmetechnik hängt von der Indikation und der institutionellen Präferenz ab:

- Dunn-45°-Projektion: sensitivste Aufnahme zur Detektion der Cam-Deformität. Durchführung: 45° Beugung und 20° Abduktion bei neutraler Rotation [21]; Zentralstrahl auf Femurkopf.
- Lauenstein-Aufnahme zur Beurteilung des anterioren und posterioren Schenkelhalses: bei ECF, da das Ausmaß des Abrutschens besonders gut beurteilt werden kann. Durchführung: Rückenlage, Bein 45° gebeugt und 45° abduziert; Zentralstrahl auf Femurkopf.
- Faux-profil-Aufnahme [22]: Darstellung der anterioren Überdachung, der Spina iliaca anterior inferior und des posterioren Gelenkspalts (Contre-coup-Läsion). Durchführung: Aufnahme im Stehen, Becken 65° zur Platte geneigt, Fuß parallel zur Platte; Zentralstrahl auf Femurkopf.

Um Beinachsendeformitäten oder Beinlängendifferenzen zu erfassen, kann eine Ganzbeinaufnahme ergänzt werden.

**Figure 4:**
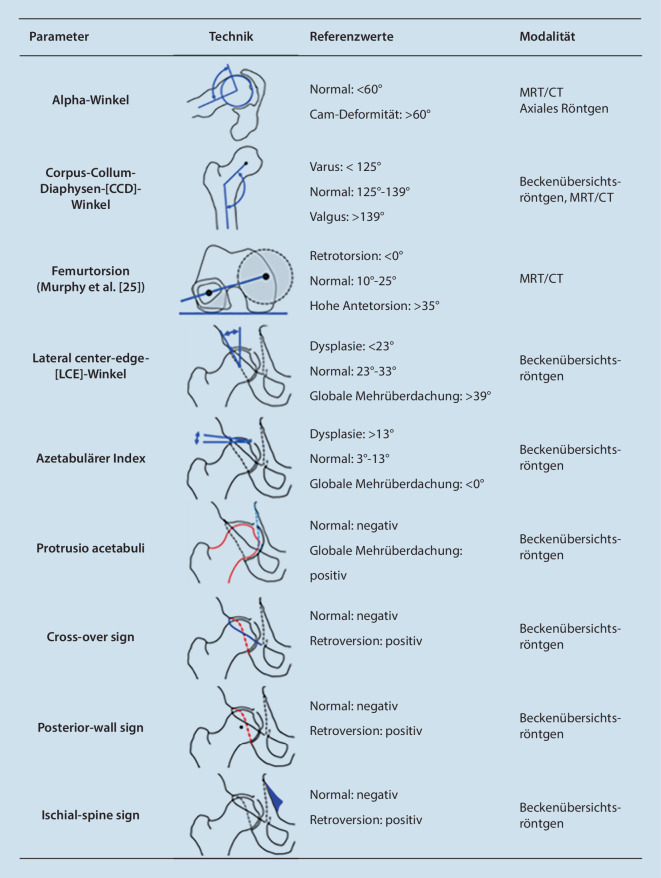

Wichtige Parameter der Projektionsröntgenaufnahme mit Normwerten zeigt (Abb. 4; [23]).

### Magnetresonanztomographie

**Figure 5:**
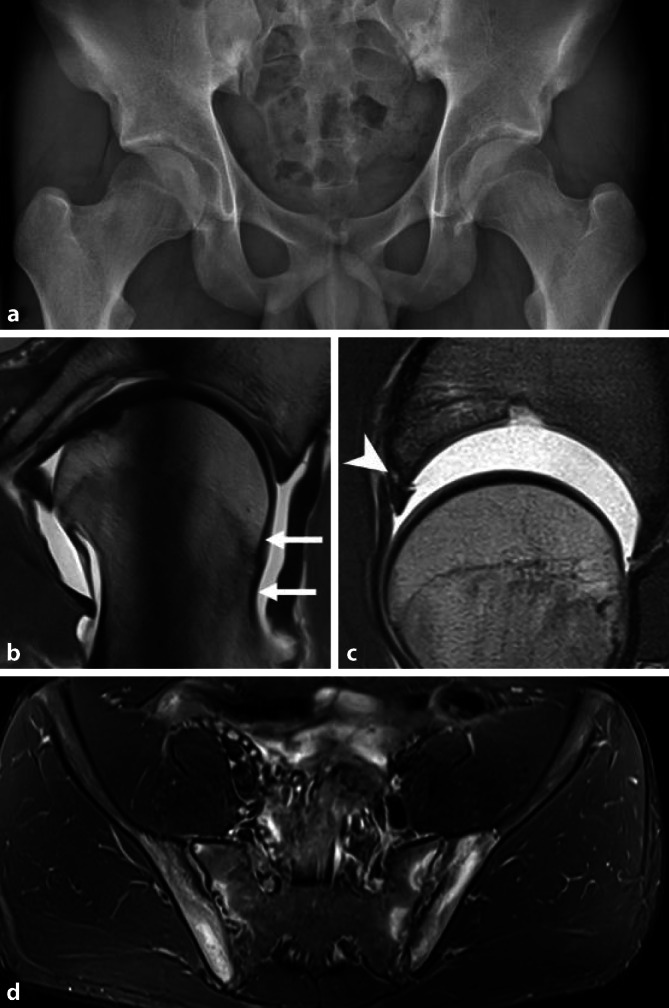

Die MRT ist die Methode der Wahl zur Darstellung chondrolabraler Läsionen und wichtig für die Therapieplanung. Ebenso können die Wachstumsfuge, osteophytäre Anbauten, reaktive Knochenmarkveränderungen, periartikuläre Weichteilalterationen als auch der Zustand der Muskulatur sensitiv erfasst werden. Der exzellente Weichteilkontrast der MRT ist insbesondere beim unreifen Skelett entscheidend, da die Cam-Morphologie teilweise knorpelig angelegt sein kann [24]. Die Feldstärke sollte zumindest 1,5 T betragen. Radiäre Turbo-Spin-Echo(TSE)-Sequenzen (oder alternativ radiäre Rekonstruktionen von 3D-Isovoxel-Sequenzen, jeweils in mindestens 12 Schichten) erlauben eine orthograde Darstellungen des Azetabulums, des Hüftkopfes und des Schenkelhalses über die gesamte Zirkumferenz [25]. Die Bezeichnung der Lokalisation folgt dabei der von Erwachsenen (Zifferblatt der Uhr, 3 Uhr entspricht beidseits jeweils anterior). Während beim Erwachsenen und Adoleszenten zur besseren Beurteilung insbesondere der Knorpeldelamination und prognostisch relevanten ausgedehnten Knorpelschäden die MR-Arthrographie in Kombination mit Traktion (bei der Traktion wird während der MRT ein dem Körpergewicht angepasster Zug auf die betroffene Hüfte ausgeübt) eine hohe Sensitivität aufweist [26], wird bei Kindern oft auf eine intraartikuläre Injektion verzichtet. Im Protokoll enthalten sein sollten jedenfalls hochaufgelöste Sequenzen über die betroffene Seite, und auch eine flüssigkeitssensitive Sequenz über das gesamte Becken zum Nachweis oder Ausschluss extraartikulärer Pathologien ([20]; Abb. 5).

Eine zusätzliche Bestimmung der azetabulären Version und femoralen sowie tibialen Torsion ist sinnvoll [15] und mit geringem Aufwand möglich [20]. Ausreichend dafür sind bei fixierten Füssen mit senkrechter Großzehe schnelle Sequenzen ohne hohe Ortsauflösung über Hüft‑, Knie- und Sprunggelenke. Bei der Bestimmung der Femurtorsion ist es von entscheidender Bedeutung, eine standardisierte Messmethode zu verwenden und diese auch entsprechend zu beschreiben, da sich die gewonnenen Messwerte beim selben Patienten je nach Messmethode um bis zu 20° unterscheiden können [17, 27]. Eine weitverbreitete Methode, die auch in den Kliniken der Autoren zur Anwendung kommt, ist die Methode nach Murphy (Messung auf Höhe des Trochanter minor; Abb. 3), da sie den Rotationsfehler auch bei Hüften mit exzessiv erhöhter Femurtorsion und Valgusdeformitäten am genauesten abbildet [28].

Eine systematische Beschreibung der Pathologien in der MRT sollte umfassen:

- *Formalterationen des proximalen Femurs und des Azetabulums*:
  *Femur: *Formalterationen des Femurkopfes, Kopf‑/Hals-Taillierung des Femurs mit Alphawinkel und Angabe der Lokalisation nach dem Zifferblatt der Uhr (12 Uhr = superior, 3 Uhr = anterior), „Herniation pit“
  *Azetabulum:* Ausmaß der Überdachung nach dem Zifferblatt, Spornbildungen, Os acetabuli
- *Labrumpathologien*: Größe des Labrums (Hypertrophie als Instabilitätszeichen vs. Hypotrophie als Hinweis auf ein Impaktions-Impingement), Intrasubstanzrisse oder Intersubstanzrisse mit chondrolabraler Separation, intralabrale oder paralabrale Ganglien, Labrumossifikation, Os acetabuli
- *Knorpel‑/Knochenschäden*: Knorpeldelamination, Dickenverlust des Knorpels, subchondrale Knochenmarködemäquivalente oder Zysten und Osteophyten, zentrale Osteophyten um die azetabuläre Fossa bzw. die Fovea capitis sowie Knorpelschäden > 2 h auf dem Ziffernblatt sind negative Prädiktoren für den langfristigen Gelenkerhalt.
- *Angrenzende/Begleitpathologien*: Zysten, ossäre Stressreaktionen am Schenkelhals/Schambeinästen, Zustand des Lig. teres, Kapseldehiszenzen oder -verwachsungen nach Voroperation, posttraumatische Verknöcherungen am indirekten Ursprung des M. rectus femoris
- *Differenzialdiagnosen/andere Veränderungen* (s. unten)
- *Beschreibung der Achsen in der Rotations-MRT:* azetabuläre Anteversion, femorale Torsion nach Murphy, (ggf. zusätzlich nach Reikeras), jeweils bilateral

## Radiologische Befunde beim FAIS

Die Befundung sollte entsprechend der rezenten ESSR-Richtlinien erfolgen [29].

### Cam-Impingement (Abb. 6)

Der anterosuperiore Kopf-Hals-Übergang ist in der Dunn-45°-Projektion besonders gut beurteilbar. Typisch sind als Ausdruck verminderte Kopf-Hals-Taillierung ein vergrößerter Alphawinkel (initiale Beschreibung durch Nötzli [30]) sowie ein verminderter Head-neck-Offset. Allerdings ist eine Bestimmung der Werte über die gesamte Zirkumferenz nur mittels MRT möglich (bzw. falls MRT kontraindiziert mittels CT). In der MRT ist ein vergrößerter femoraler Alphawinkel über 60° typischerweise anterosuperior sichtbar [31]. Der mechanische Konflikt mit dem Azetabulum findet beim Cam-Impingement in der Gelenkperipherie statt. Ausgehend von einer Läsion in der chondrolabralen Übergangszone kommt es zu einer teppichartigen Delamination des azetabulären Knorpels von peripher nach zentral (Outside-in-Läsion im Gegensatz zur Inside-out-Läsion bei der Hüftdysplasie) und Ablösung des Labrums vom azetabulären Erker ([32]; Abb. 1 und 2). Charakteristische assoziierte Läsionen sind ein Os acetabuli sowie am Schenkelhals das Vorliegen einer kleinen randsklerosierten Zyste, dem sog. „herniation pit“, die typischerweise am punctum maximum der Cam-Deformität auftreten [1].

**Figure 6:**
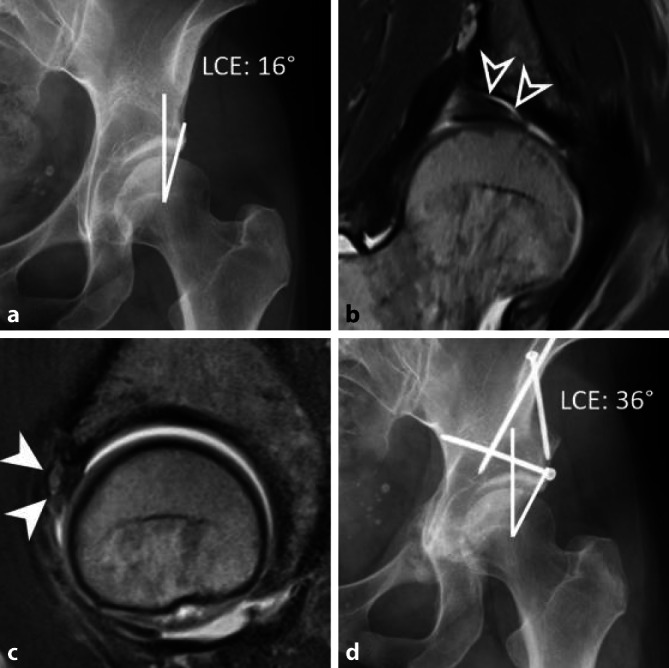

Bei Adoleszenten mit FAI sind Knorpelschäden mit 88,4 % sehr häufig und korrelieren in ihrem Ausmaß mit dem Alphawinkel [33]. Dabei weisen Patienten mit Post-ECF-Slip Deformitäten im Vergleich zum primären Cam-FAI häufig einen höheren Alphawinkel und ausgedehntere Knorpelschäden auf; zudem sind die Patienten im Schnitt jünger [34].

### Pincer-Impingement (Abb. 6)

Die azetabuläre Retroversion stellt ein Spektrum von wenig bis ausgeprägter Malrotation des Hemipelvis und Azetabulums dar, welches in erster Linie konventionell radiologisch beurteilt wird. Am häufigsten (bis zu 80 %) liegt ein kraniales Cross-over-Zeichen (COS) mit normaler Anteversion der Pfanne auf Höhe des Femurkopfzentrums vor (Prävalenz bis zu 80 %). Das COS ist positiv, wenn in der a.‑p.-Projektion der hintere Pfannenrand den vorderen Pfannenrand kreuzt (Abb. 4). Mit zunehmendem Schweregrad nimmt die kraniokaudale Ausdehnung des retrovertierten Azetabulums (kleiner als ein Drittel) zu, und es finden sich zudem ein positives Posterior-wall- und Ischial-spine-Zeichen in der a.‑p.-Projektion. Das Posterior-wall-Zeichen ist positiv, wenn der Hüftkopfmittelpunkt sich lateral des hinteren Pfannenrands projiziert, das Ischial-spine-Zeichen, wenn die Spina ischiadica am medialen Rand des kleinen Beckens sichtbar ist (Abb. 4). Bei bis zu 15 % der Patienten liegen Kombinationen dieser Retroversionszeichen als Ausdruck einer globalen Retroversion vor [35].

Eine vermehrte azetabuläre Überdachung kann entweder global oder fokal bestehen. In der Beckenübersichtsaufnahme sind die wichtigsten Messparameter der Lateral-Center-Edge(LCE)-Winkel, der bis zum lateralen Ende der Sklerosezone gemessen werden sollte (da dies dem gewichtstragenden Anteil des Azetabulums entspricht; Abb. 6) sowie der azetabuläre Index [36]. Diese Winkel stellen robuste Parameter dar, die nicht oder nur gering lage- und verkippungsabhängig sind [36]. In der Routinediagnostik gelten als Kriterien für eine globale Mehrüberdachung ein LCE-Winkel von ≥ 40° oder eine negative azetabuläre Inklination (azetabulärer Index < 0). Die Protrusionshüfte stellt die Extremform der globalen Pincer-Morphologie dar.

Die Mehrüberdachung führt zu in der MRT sichtbaren Rissen in der Substanz des Labrums; charakteristischerweise sieht man häufig ein hypotrophes, degeneriertes Labrum oder ossäre Metaplasien, und eher schmale, zirkumferente Knorpelschäden [37]. Bei Vorliegen einer globalen Mehrüberdachung kommt es durch die Fehlbelastung zu einer Chondropathie medial sowie durch eine sekundäre posteroinferiore Translation des Femurkopfes zu Knorpelschäden ebenda (*Contre-coup-Läsion*; [32]).

## Differenzialdiagnosen

Die wichtigste Differenzialdiagnose der ossären Veränderungen beim FAIS stellt die Hüftdysplasie dar, welche mittels einer Beckenosteotomie behandelt wird. (Abb. 6). Extraartikuläre Ursachen für ein Impingement-Syndrom der Hüftregion umfassen das subspinale Impingement nach Avulsionsfrakturen oder Apophysenverletzungen und das ischiofemorale Impingement, Letzteres typischerweise bei Coxa valga et antetorta [38]. Auch die häufige Coxa saltans interna (das sog. Psoasschnappen durch mechanische Irritation der Psoassehne in ihrem Verlauf über den vorderen Pfannenrand) oder Stressfrakturen sind als Differenzialdiagnosen bedeutsam. Anteriorer Hüftschmerz kann zudem durch lumbale oder Schmerzen im Iliosakralgelenk (ISG), eine Leistenhernie oder abdominelle Erkrankungen vorgetäuscht werden. Ebenso müssen andere muskuloskeletale Ursachen, wie beispielsweise Entzündungen oder Muskelverletzungen, ausgeschlossen werden ([39]; Abb. 5). Zu beachten ist, dass bei Patienten mit Sportlerhernie, Adduktorenschmerz oder überlastungsbedingter Pubalgie häufig konkomitant ein FAI besteht [40].

## Fazit für die Praxis

- Das femoroazetabuläre Impingement (FAI) ist ein schmerzhaftes Syndrom des Hüftgelenks, verursacht durch einen repetitiven mechanischen Konflikt zwischen Azetabulum und proximalem Femur.
- Verantwortlich sind Taillierungsstörungen des Femurs (Cam), eine Mehrüberdachung und/oder Retroversion des Azetabulums (Pincer) oder Mischformen; ein wichtiger Einflussfaktor ist die Femurtorsion.
- Das Cam-Impingement entsteht typischerweise durch eine pathologische Belastung während des Wachstumsschubs, oder sekundär beispielsweise nach Epiphyseolysis capitis femoris (ECF) oder M. Perthes.
- Initial sollten eine Beckenübersichtsaufnahme und eine axiale Röntgenaufnahme durchgeführt werden; wichtige Parameter sind der Lateral-Central-Edge(LCE)-Winkel, das Cross-over‑, Posterior-wall- und das Ischial-spine-Zeichen.
- Die Magnetresonanztomographie (MRT) dient der genauen Beurteilung von Formalterationen (wichtiger Parameter bei der Beurteilung des Cam-Impingements ist der Alphawinkel) und der Beurteilung des Labrums, des Knorpels und subchondralen Knochens sowie von Begleitpathologien und Differenzialdiagnosen; axiale Sequenzen (*Rotations-MRT*) erlauben die Bestimmung der Azetabulumversion und der Torsion der unteren Extremität.
